# Transcription of hexose transporters of *Saccharomyces cerevisiae *is affected by change in oxygen provision

**DOI:** 10.1186/1471-2180-8-53

**Published:** 2008-03-28

**Authors:** Eija Rintala, Marilyn G Wiebe, Anu Tamminen, Laura Ruohonen, Merja Penttilä

**Affiliations:** 1 VTT, Technical Research Centre of Finland, P.O. Box 1000, FI-02044 VTT, Finland

## Abstract

**Background:**

The gene family of hexose transporters in *Saccharomyces cerevisiae *consists of 20 members; 18 genes encoding transporters (*HXT1-HXT17*, *GAL2*) and two genes encoding sensors (*SNF3*, *RGT2*). The effect of oxygen provision on the expression of these genes was studied in glucose-limited chemostat cultivations (D = 0.10 h^-1^, pH 5, 30°C). Transcript levels were measured from cells grown in five steady state oxygen levels (0, 0.5, 1, 2.8 and 20.9% O_2_), and from cells under conditions in which oxygen was introduced to anaerobic cultures or removed from cultures receiving oxygen.

**Results:**

The expression pattern of the *HXT *gene family was distinct in cells grown under aerobic, hypoxic and anaerobic conditions. The transcription of *HXT2*, *HXT4 *and *HXT5 *was low when the oxygen concentration in the cultures was low, both under steady state and non-steady state conditions, whereas the expression of *HXT6*, *HXT13 *and *HXT15/16 *was higher in hypoxic than in fully aerobic or anaerobic conditions. None of the *HXT *genes showed higher transcript levels in strictly anaerobic conditions. Expression of *HXT9*, *HXT14 *and *GAL2 *was not detected under the culture conditions studied.

**Conclusion:**

When oxygen becomes limiting in a glucose-limited chemostat cultivation, the glucose uptake rate per cell increases. However, the expression of none of the hexose transporter encoding genes was increased in anaerobic conditions. It thus seems that the decrease in the moderately low affinity uptake and consequently the relative increase of high affinity uptake may itself allow the higher specific glucose consumption rate to occur in anaerobic compared to aerobic conditions.

## Background

The hexose transporter gene family in *Saccharomyces cerevisiae *contains the sugar transporter genes *HXT1 *to *HXT17, GAL2 *and the glucose sensor genes *SNF3 *and *RGT2 *[1,2]. The proteins encoded by *HXT1 *to *HXT4 *and *HXT6 *to *HXT7 *are considered to be the major hexose transporters in *S. cerevisiae*. The MC996A yeast strain lacking these six transporters and the protein encoded by *HXT5 *(referred to as a *hxt *null mutant) is unable to grow on glucose [3]. Based on their K_m _values and their ability to restore growth on glucose when the respective genes are expressed individually in the *hxt *null mutant strain, these transporters have been classified as high (Hxt6p, Hxt7p), moderately low (Hxt2p, Hxt4p) and low (Hxt1p, Hxt3p) affinity transporters. However, Hxt2p in cells grown on low glucose concentrations exhibits both high and low affinity transport kinetics [4]. Gal2p is able to transport glucose with high affinity but the gene encoding it is expressed only when galactose is present [4,5]. In the CEN.PK2-1C strain, deletion of only *HXT1 *to *HXT7 *is not enough to completely abolish growth on glucose, fructose or mannose [6]. Only deletion of all seventeen of the *HXT *genes and *GAL2 *produces a strain unable to grow on these sugars, and overexpression of any individual gene encoding a hexose transporter, except *HXT12 *in this multiple deletant restores growth on at least one of the hexoses: glucose, fructose, mannose or galactose [6].

The major glucose transporters are regulated at transcriptional level by the extracellular glucose concentration [7]. The transcription factor Rgt1p represses the genes encoding these transporters in the absence of glucose, but it is also required for full induction of *HXT1 *on high levels of glucose [8]. Paralogous proteins Mth1p and Std1p are necessary for Rgt1p to act as a repressor. Release of the repression requires removal of Mth1p and Std1p as well as phosphorylation of Rgt1. The glucose signal mediated by the Snf3p and Rgt2p sensors stimulates the degradation of Mth1p and Std1p, while the glucose signal mediated by the G-protein-coupled receptor Gpr1p leads to activation of protein kinase A, which phosphorylates Rgt1 and releases the repression of *HXT *genes [9-15]. In addition, high affinity transporters are repressed in high levels of glucose via the Snf1p-Mig1p glucose repression pathway. Mig1p binds directly to the promoters of *HXT2 *and *HXT4 *and also represses the expression of *MTH1 *and *SNF3 *[15,16].

Expression of *HXT5 *is regulated by growth rate rather than the external glucose concentration [17,18]. It is expressed upon decrease in the growth rate of cells in glucose batch cultivations, at low dilution rates in glucose-limited chemostat cultivations, on non-fermentable carbon sources and during sporulation [17,19]. The regulation is mediated by STRE and HAP elements in the promoter region of *HXT5*. The STRE elements are needed for induction at low growth rates and during growth on ethanol, and the HAP elements for growth on ethanol or glycerol [17]. The Hxt5p transporter shows moderately low affinity for glucose [19].

The hexose transporters encoded by *HXT8 *to *HXT17 *have not been studied to the same extent as the major *HXT *genes. Many of the functions assigned to these transporters do not directly relate to sugar utilisation. Diderich and co-workers [20] were not able to detect the expression of these genes when probing total RNA from glucose-limited chemostat cultivations. It is known, however, that the regulation of all but *HXT11 *and *HXT12 *is controlled by glucose [7]. The transporters encoded by *HXT9 *and *HXT11 *are also shown to be involved in pleiotropic drug resistance [21], and the promoter of *HXT17 *is a target for Mac1p transcription factor, which regulates high-affinity copper uptake genes under copper-deficient conditions [22,23]. Greatrix and co-workers [24] demonstrated that *HXT5, HXT13 *and *HXT15 *are induced in the presence of non-fermentable carbon sources, and that *HXT17 *is upregulated when yeast cells are grown on medium containing raffinose and galactose at pH 7.7 but not at pH 4.7. In addition, Hxt9p and Hxt10p are able to transport arsenic trioxide into the cell, as do the major hexose transporters [25].

In addition to regulation at transcriptional level, inactivation of Hxt proteins takes place under certain conditions such as starvation or in the presence of high concentrations of glucose [7]. Degradation via endocytosis, autophaghy, and transport to the vacuole has been shown for Hxt2p, Hxt5p, Hxt6p and Hxt7p [26-30].

In glucose-limited chemostats, the specific glucose consumption rate is inversely related to the oxygen provided to the system [31,32]. Under oxygen-limited or anaerobic conditions, energy is provided by respiro-fermentative and fermentative metabolism, respectively. The biomass yield on glucose is lower and the cells must transport more glucose per unit time to be able to grow at the same rate in anaerobic or oxygen-limited conditions as when the metabolism is fully respirative. It is not clear what modifications in the cell are responsible for the higher glucose uptake. In addition, in spite of numerous studies, the mechanism(s) controlling glycolysis in yeast is still unknown. Control of individual steps or enzymes of this pathway, such as the phosphorylation of glucose and the control of phosphofructokinase have been extensively studied [33-35]. However, it is likely that the control is distributed over a number of steps, since the overexpression of individual glycolytic enzymes, or of all, did not enhance glycolytic flux [36-38]. Glucose transport has been suggested to play a major role in the control of glycolysis [39], and shown to be highly important for the dynamics of glycolysis with substantial control over the frequency of glycolytic oscillations [38]. Additionally, glucose transport may control the flux of glycolysis, even at high external glucose concentrations if transport capacity is reduced, as is the case in a strain that expresses a chimera between Hxt1p and Hxt7p and no other hexose transporter [40,41].

Previously, we have assessed the role of oxygen in the physiology of *S. cerevisiae *by studying the metabolite and transcript levels related to central carbon metabolism [32]. In the present study we extend the analysis to glucose transport to determine if and how hexose transporters are affected when oxygen becomes limiting for growth. We probed cells from glucose-limited chemostat cultivations of CEN.PK113-1A at five different oxygen levels for the expression of *HXT1 *to *HXT11, HXT13 *to *HXT17, GAL2, SNF3 *and *RGT2*. In addition, we studied the expression of these genes under conditions in which oxygen was removed from cultivations receiving it or introduced to anaerobic cultivations.

## Results

### Transcription of *HXT *genes in cells at steady state exposed to different oxygen levels

Five different inflow gas oxygen levels (20.9, 2.8, 1.0, 0.5 and 0%) were used to study the response in expression of genes encoding hexose transporters and glucose sensors to external oxygen in glucose-limited chemostat cultures of CEN.PK113-1A. The specific glucose consumption rates were 6.6, 8.0, 11.4, 14.3 and 37.1 Cmmol g biomass^-1 ^h^-1 ^for chemostat cultures receiving 0.5, 1.0, 2.8 and 20.9% oxygen, respectively [32]. Extracellular glucose was not detected. The strongest expression signals were obtained from *HXT6, HXT7 *and *HXT10*. The signals of *HXT9*, *HXT14 *and *GAL2 *transcripts were below the detection limit in these conditions.

**Figure 1:**
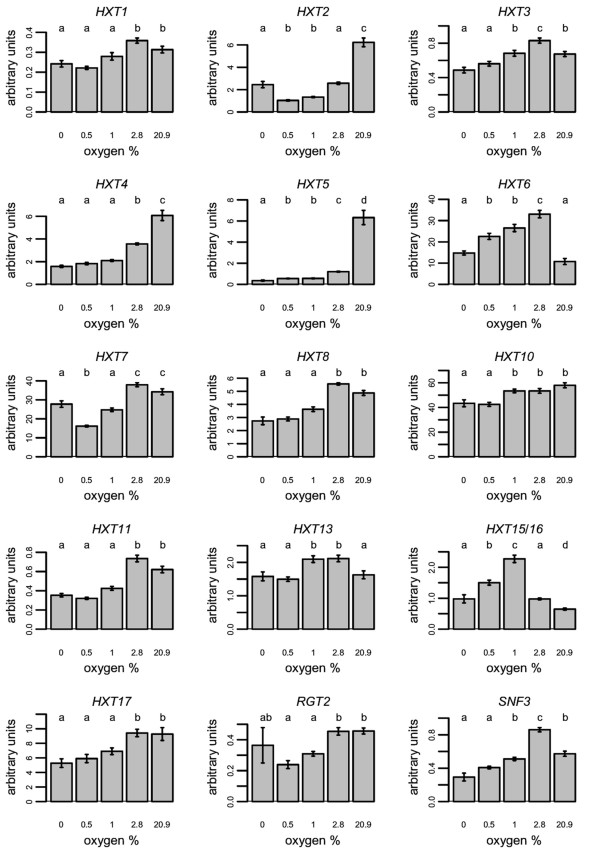
**The expression of *HXT *genes at different steady state oxygen levels**. The expression of genes encoding hexose transporters and glucose sensors in steady state oxygen concentrations of 0, 0.5, 1.0, 2.8 or 20.9% O_2 _in glucose-limited chemostats (D = 0.10 h^-1^, pH 5.0, 30°C, and 1.5 vvm gas flow). Error bars indicate ± sem for 4 to 8 samples taken during steady states in 2 to 4 cultivations. Values with the same letter (a to e) for the same gene did not differ significantly (p > 0.05, Dunnett's T3 multiple range test) from data points showing the same letter.

Statistical differences (p < 0.05) in the expression levels of all the hexose transporter encoding genes were detected between at least some of the oxygen concentrations studied (Figure 1). *HXT2, HXT4 *and *HXT5 *were significantly more highly expressed in aerobic conditions compared to hypoxic (2.8, 1.0 and 0.5% O_2_) or anaerobic conditions. Each of these genes showed some increase in expression already at 2.8% O_2_. *HXT2 *also had higher transcript levels in anaerobic conditions compared to the hypoxic conditions of 1.0 and 0.5% O_2_. *HXT1, HXT8*, *HXT11 *and *HXT17 *had higher expression in 2.8% O_2 _and fully aerobic conditions compared to lower oxygen levels, whereas *HXT10 *had higher expression at 1% or more O_2_. The signals from *HXT6*, *HXT13 *and combined *HXT15 *and *HXT16 *(*HXT15/16*) were higher in at least two out of three hypoxic conditions than in either aerobic or anaerobic conditions. The highest transcript levels of *HXT6 *and *HXT3 *were seen in 2.8% O_2_, whereas the expression of *HXT15/16 *was reduced at this level of oxygen provision. *HXT13 *had similar high expression in 1 and 2.8% O_2_. None of the hexose transporter genes were upregulated in anaerobic compared to aerobic conditions. The signal for the gene encoding the Snf3p sensor was higher in aerobic compared to anaerobic conditions, and highest in 2.8% O_2_, whereas the signal of the *RGT2 *transcripts was lowest under the hypoxic conditions of 0.5 and 1% O_2_.

### Expression of transporter encoding genes after a change between aerobic and anaerobic conditions

**Figure 2:**
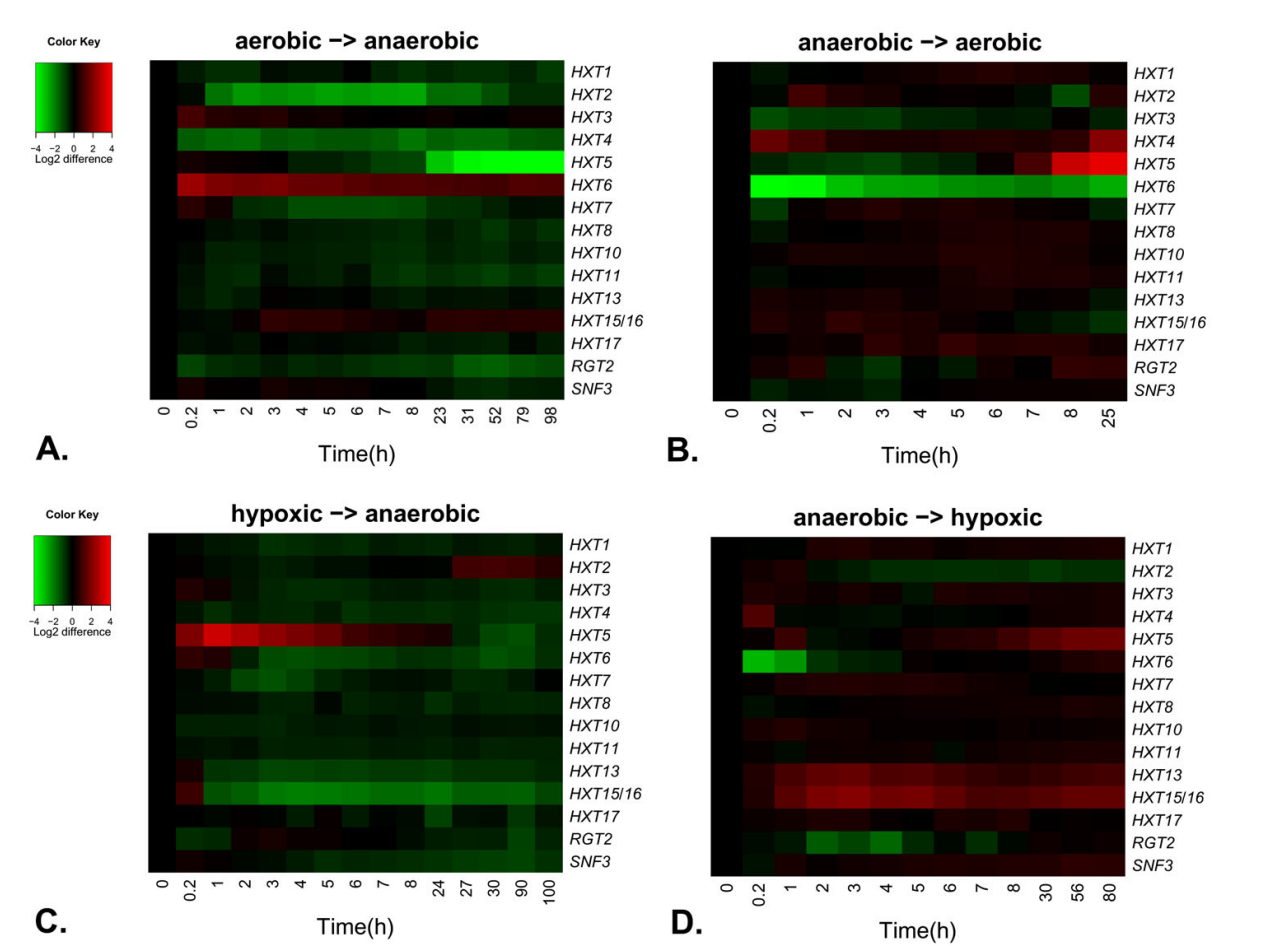
**Relative expression levels of *HXT *genes after a change in oxygen provision**. The heatmap of the relative expression levels of genes encoding hexose transporters and glucose sensors after change in oxygen provision. A. Shift from aerobic to anaerobic conditions. B. Shift from anaerobic to aerobic conditions. C. Shift from hypoxic (1% O_2_) to anaerobic conditions. D. Shift from anaerobic to hypoxic (1% O_2_) conditions.

The expression of hexose transporter and glucose sensor genes following a change in oxygen provision was studied by switching oxygen provision off or on, in aerobic and anaerobic cultivations, respectively. Following the change from aerobic to anaerobic conditions, samples were taken until a new steady state was achieved (Figure 2A). After the switch from anaerobic to aerobic conditions, the cultivations started to oscillate after 25 hours and the steady state could not be reached. The results are thus reported only for the first 25 hours (Figure 2B).

**Figure 3:**
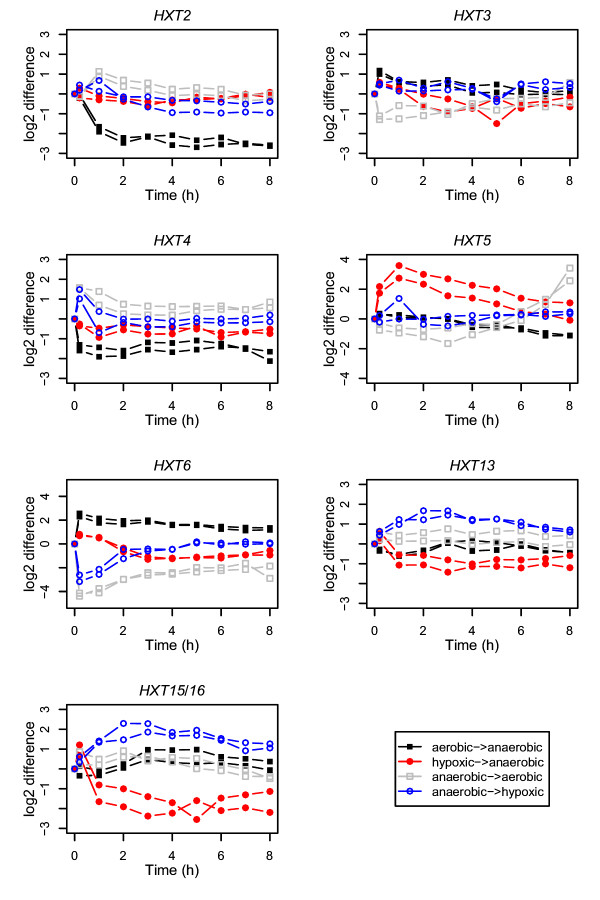
**Initial responses of specific *HXT *genes to the change in oxygen concentration**. The relative expression of *HXT2*-*HXT6*, *HXT13 *and *HXT15/16 *during the first eight hours after the switch in oxygen concentration. Symbols with the same colours represent replicate cultivations carried out in the same conditions.

Clear changes as a response to the removal of oxygen from respiratory cells were seen in the transcript levels of *HXT2 *to *HXT7*, although the time scales of the responses varied (Figures 2A and 3). Two of these genes (*HXT3 *and *HXT6) *were upregulated and four (*HXT2, HXT4*, *HXT5 *and *HXT7) *downregulated either transiently or permanently as a response to lack of oxygen. The transcript levels of *HXT3, HXT4, HXT6 *and *HXT7 *had changed already during the first 10 minutes, while a response in *HXT2 *expression was seen only after 10 min but within one hour. Changes observed in *HXT5 *expression were the slowest to occur; a significant response was seen after 6 hours.

When oxygen was introduced to the anaerobic cultivations (Figures 2B and 3), two of the genes encoding major hexose transporters (*HXT3 *and *HXT6) *were downregulated and two (*HXT2 *and *HXT4) *were upregulated. *HXT5 *was upregulated only after a transient downregulation during the first 4 hours after oxygen was provided. *HXT7 *was slightly upregulated after initial (10 min after oxygen was provided) downregulation. Expression of *HXT3, HXT4*, *HXT6 *and *HXT7 *exhibited rapid responses to a change in environmental oxygen, i.e within 10 min, the earliest sample taken after the change.

The responses of the transporter genes *HXT8 *to *HXT17 *were weaker than those of the major hexose transporters (Figures 2A, 2B and 3). In general, these genes were upregulated when oxygen was introduced to anaerobic cultivations, and either downregulated or did not respond when oxygen was removed from aerobic cultures. The expression of *HXT15/16*, increased only transiently when the culture was adapting to aerobic conditions and permanently following the shift to anaerobic conditions. Of the sensor genes, expression of *SNF3 *was not affected by change in the oxygen concentration, but *RGT2 *was downregulated when the cultures experienced lack of oxygen.

### Expression of transporter encoding genes after a change between hypoxic and anaerobic conditions

The response in expression of *HXT *and glucose sensor genes was also assessed following a shift from hypoxic (1% O_2_) to anaerobic and from anaerobic to hypoxic conditions. Samples were taken until a new steady state was achieved.

The response in the expression of the hexose transporter encoding genes to oxygen depletion was different when the starting point was hypoxic and not fully aerobic (Figures 2C–D and 3). When oxygen was replaced with nitrogen in the cultures which had been hypoxic, most of the *HXT *genes were downregulated (Figure 2C). The expression of *HXT3, HXT4, HXT6*, *HXT13 *and *HXT15/16 *was transiently upregulated in cells after 10 min in anaerobic conditions, but downregulated already after 60 min. Only *HXT5 *was transiently upregulated for a longer period (the first 7 hours). *HXT2 *was upregulated towards the end of the adaptation to the anaerobic conditions, around 30 hours after oxygen was depleted.

When hypoxic conditions were introduced to anaerobic cultivations (Figure 2D) the immediate (10 min) responses were similar to those observed when fully aerobic conditions were introduced (Figure 2B), but the subsequent responses differed. Only the expression of *HXT2, HXT5, HXT13 *and *HXT15/16 *changed significantly as the cells approached the hypoxic steady state. *HXT5, HXT13 *and *HXT15/16 *were upregulated whereas *HXT2 *was downregulated. *HXT6 *and *RGT2 *were transiently downregulated during the first 2 and 2–5 hours, respectively, following the switch to hypoxic conditions.

### Promoter analysis of the *HXT *genes induced in hypoxic conditions

The promoter regions (950 bp upstream from the start codon) of the genes encoding Hxt3p, Hxt6p, Hxt13p, and Hxt15/16p transporters were sequenced in the CEN.PK113-1A strain. The upstream sequences of *HXT3, HXT6 *and *HXT15/16 *were identical to those of strain S288C, whose genomic sequence is found in the data banks. In the promoter region of *HXT13 *there were five separate nucleotides in CEN.PK113-1A that differed from the published sequence of strain S288C. The DNA Pattern [42] web-based tool was used to search the promoters of *HXT3*, *HXT6, HXT13 *and *HXT15/16 *for binding sites of transcription factors known to be involved in hypoxic control. These were LORE (low oxygen response element); 5' ACTCAACAA 3' [43] and HRE (hypoxic response element); 5' BRCGTGVBBB 3' [44]. Two LORE sequences were found in the *HXT3 *promoter and one in the *HXT15/16 *promoter (one mismatch allowed), whereas one HRE site was found in the *HXT13 *promoter of both S288C and CEN.PK113-1A strains.

## Discussion

Most of the published data indicate that the expression of the hexose transporter encoding genes of *S. cerevisiae *is mainly regulated by extracellular glucose concentration. In the present paper, the expression of these genes was studied under various oxygen concentrations in glucose-limited chemostats with an extracellular glucose concentration below detection limit and thus not affecting the transcription of *HXT *genes [20]. When oxygen becomes limiting in a glucose-limited chemostat cultivation, the glucose uptake rate per cell increases. We addressed the question of how the expression level of hexose transporter encoding genes is modified as a result of increased specific glucose consumption rate under oxygen restricted conditions.

In aerobic glucose-limited chemostat cultures grown at a dilution rate of 0.1 h^-1^, both high and moderately low affinity glucose uptake is detected [20]. A similar result was observed here; expression of the moderately low affinity transporter genes *HXT2, HXT4 *and *HXT5 *and of the high affinity transporter genes *HXT6 *and *HXT7 *was seen. Expression of *HXT1 *and *HXT3*, encoding low affinity transporters was also detected, but on a very low level. In their chemostat study, Diderich and co-workers [20] only detected the expression of *HXT2, HXT5 *and *HXT7*. This is most probably due to differences in the sensitivity of the techniques used. We were also able to detect the expression of all other hexose transporter genes except *HXT9, HXT14 *and *GAL2*, although some were only expressed at a very low level.

In anaerobic conditions, the expression of *HXT2, HXT4 *and *HXT5*, encoding moderately low affinity transporters was significantly reduced compared to aerobic conditions (Figure 1). This was also seen during transition from aerobic or hypoxic growth to anaerobic growth and agrees with earlier studies which detected only high affinity transport activity in anaerobic glucose-limited chemostat cultivations [20]. In addition to the decreased expression levels of the moderately low affinity transporter encoding genes, the activity of Hxt2p may be modulated towards higher affinity, as both high and moderately low affinity components have been observed in a strain expressing only this transporter gene [4].

Interestingly, the expression of none of the hexose transporter encoding genes was increased in anaerobic compared to aerobic conditions. However, the decrease in the moderately low affinity uptake may result in a relative increase in high affinity uptake, which may allow the higher specific glucose consumption rate to occur in anaerobic conditions. In contrast, *Trhxt1*, encoding a glucose transporter, is induced by anoxia but not by hypoxia in the filamentous fungus *Trichoderma reesei *[45]. This may represent a difference between facultative anaerobic and strictly aerobic fungi.

Earlier studies have shown that *HXT5 *is expressed at a higher level in aerobic than in anaerobic conditions [20,46]. Our studies show that the transcription of *HXT5 *was significantly reduced not only in anaerobic, but also in hypoxic, compared to aerobic conditions. In addition, the regulation of *HXT5 *following a change in oxygen provision differed from that of other *HXT *genes. When hypoxic condition became anaerobic, there was a long (7 h), transient upregulation of expression of this gene, that was opposite to the final response of downregulation. It is known that the transcription of *HXT5 *is not regulated by extracellular glucose concentration, but by growth rate, and that the expression is under the control of STRE and HAP elements in its promoter [17,19]. The reduction in specific growth rate for up to 15 h following a shift from hypoxic to anaerobic conditions may have contributed to the transient upregulation of *HXT5*, but it should be noted that transient upregulation was not observed following the change from aerobic to anaerobic conditions even though the specific growth rate was similarly reduced. HAP2/3/4/5p elements are needed for the induction of transcription of respiratory genes, like the ones encoding the subunits of cytochrome *c *oxidase (*COX4, COX5a, COX6*) and enzymes of the tricarboxylic acid cycle (*KGD1, CIT1*) [47-51]. These elements are also likely to be involved in the aerobic induction of *HXT5 *seen in this study.

The change in specific growth rate after a change in oxygen provision may also have affected the expression of other hexose transporter encoding genes, directly or inderectly. However, responses to increased oxygen provision (20.9 or 1.0%) which occurred within less than 2 hours were independent of specific growth rate, which only increased after this time. Further, specific growth rate increased 2 hours after a change to either 20.9 or 1.0% oxygen, but for those genes whose transcription was affected by the change between 2 and 8 hours after it occurred, during which time the specific growth rate was increased, the response was generally not the same with 20.9 as with 1.0% oxygen. Thus it is unlikely that the increase in specific growth rate contributed as much as the increase in oxygen availability in regulation of the hexose transport genes. The decrease in specific growth rate which occurred following a change to anaerobic conditions was also unlikely to be as important as the reduction in oxygen availability in affecting transcription of the hexose transport genes, since a similar reduction in specific growth rate was observed regardless of the initial concentration of oxygen provided, but changes in gene transcription were dependent on the initial oxygen provision.

In hypoxic (0.5–2.8% oxygen) conditions, the expression of four of the *HXT *genes was higher than in aerobic conditions. Even with 2.8% oxygen, compared to aerobic conditions, the expression of *HXT3, HXT6, HXT13 *and *HXT15/16 *was higher, whereas the expression of *HXT2, HXT4 *and *HXT5 *was lower. In our earlier study we observed that the expression levels of 50% of the 69 genes of central carbon metabolism which were studied were different under 2.8% oxygen provision compared to full aeration [32], suggesting that the cell is able to recognise the reduced oxygen level, even though the cultures still maintain a high biomass concentration and a high oxygen uptake rate. Reduced transcript levels of *HXT2 *and *HXT5 *and increased transcript levels of *HXT3 *have previously been observed during respiro-fermentative metabolism at high dilution rates in chemostat cultures [20].

The expression of the high affinity transporter encoding gene *HXT6 *was relatively strong in all the hypoxic oxygen levels studied compared to aerobic or anaerobic conditions. This was seen in both steady state and non-steady state cultures. In contrast, the expression of the other high affinity transporter encoding gene, *HXT7 *was very similar in aerobic cultivations and in those receiving 2.8% oxygen and was low in cultures with less or no oxygen, especially in the culture receiving 0.5% oxygen. This observation agrees with the earlier observation that even though *HXT6 *and *HXT7 *are 99% identical in their coding regions, they are not co-regulated [20] Indeed, their promoter regions are only 51% identical.

*HXT13 *and *HXT15/16 *exhibited their highest relative expression in hypoxic steady states and also showed clear responses to a shift in oxygen concentration. These genes were induced when hypoxia was introduced after anaerobic conditions and repressed when hypoxic cultivations became anaerobic. The transient responses following shifts between fully aerobic and anaerobic conditions were smaller, reflecting the comparable expression level of these two genes under the two conditions. *HXT13 *and *HXT15 *have previously been shown to be slightly induced by non-fermentable carbon sources [24]. In the promoter regions of *HXT15 *and *HXT16 *there are LORE (low oxygen response element) [43] sequences with one mismatch to the consensus. These elements could be responsible for the induction in the hypoxic conditions, although the site is located -900 bp from the start codon of the genes. The promoter of *HXT13*, on the other hand, contains an HRE (hypoxia response element) sequence, which is responsible for the regulation of hypoxic genes in mammalian cells (*e.g. GLUT1 *glucose transporter in human) [52-55]. It has recently been speculated that this element is also present in the yeast genome [44].

Only small differences in the expression levels of *HXT8, HXT10, HXT11 *and *HXT17 *were seen, either between different steady states or as a response to a change in oxygen concentration. In addition to regulation by glucose concentration (excluding *HXT11)*, the expression levels are known to be affected by change in pH (*HXT8*, *HXT9, HXT11 *and *HXT17*), and *HXT11 *has been indicated to be involved in multidrug resistance [7,24,56].

Most of the *HXT *genes had different expression in different oxygen concentrations, either during the steady states or following a change between anaerobic and hypoxic or aerobic conditions. Transcription of *HXT2, HXT4 *and *HXT5 *was high in aerobic and that of *HXT6, HXT13 *and *HXT15/16 *was high in hypoxic conditions. Additionally, the three former transporter encoding genes clearly had lower expression levels in oxygen-restricted conditions. However, questions concerning the significance of the hypoxic induction, and why the moderately low affinity transporters are expressed in glucose-limited chemostats with very low extracellular glucose concentration, remain to be addressed in future studies. It is tempting to speculate, that the hypoxic induction of expression, in particular that of *HXT6*, encoding a known high affinity transporter, indicates a response to enhance sugar uptake upon oxygen limitation. The fact that the relative transcript levels of this gene are lower in fully anaerobic compared to hypoxic cultures may not represent the situation at the protein level on the plasma membrane. The decreased amount of moderately low affinity transporters, achieved by both regulating the expression of these genes and by degradation of the respective protein(s), under conditions of low oxygen may provide additional membrane space for the high affinity transporters to occupy the membrane and thus increase the glucose uptake rate.

## Conclusion

The expression of hexose transporter encoding genes was affected by change in oxygen provision. The expression of genes encoding moderately low affinity transporters was lower in anaerobic than aerobic conditions. As the expression of none of the hexose transporter encoding genes was increased in anaerobic compared to aerobic conditions it seems that the decrease in the moderately low affinity uptake and consequently the relative increase of high affinity uptake may itself allow a higher specific glucose consumption rate to occur in oxygen restricted and anaerobic conditions. Further, the gene encoding the high affinity transporter Hxt6p and the genes encoding Hxt13p and Hxt15/16p were upregulated in hypoxic conditions, and the expression of moderately low affinity transporters, particularly that of *HXT5*, was reduced with only 2.8% oxygen compared to fully aerobic conditions. The regulation of hexose transporter encoding genes is thus different in oxygen restricted conditions compared to fully anaerobic conditions.

## Methods

### Yeast strain and culture conditions

*Saccharomyces cerevisiae *CEN.PK113-1A (*MAT*α, *URA3*, *HIS3*, *LEU2*, *TRP1*, *MAL2-8c*, *SUC2) *was grown in 0.8 to 1 L medium in Braun Biotech International (Sartorius) Biostat^® ^CT (2.5 L working volume) bioreactors in the defined minimal medium described by Verduyn *et al*. [57], with 10 g glucose l^-1 ^as carbon source, and supplemented with 10 mg ergosterol l^-1 ^and 420 mg Tween 80 l^-1^. BDH silicone antifoam (0.5 mL l^-1^) was used to prevent foam production in the cultures. Chemostat cultures were maintained at D = 0.10 ± 0.02 h^-1^, pH 5.0, 30°C, with 1.5 volume gas [volume culture]^-1 ^min^-1 ^(vvm). For cultures which received less than 20.9% O_2 _in the gas stream, air was replaced with the equivalent volume of N_2_, so that total gas flow was maintained constant for all experiments. Cultures which were fed 2.8 or 20.9% O_2 _were subject to oscillations. To prevent these, approximately 5% of the total cell concentration in the bioreactor was added to the culture as cells in mid to late exponential phase at the time when continuous medium feed was started [58]. The cultivations and the culture conditions, biomass determination and metabolite analyses are described in more detail by Wiebe and coworkers [32].

In some cultures, the steady state was disrupted by replacing air (20.9 or 1.0% O_2_) with 100% N_2 _(0% O_2_) or by introducing air (20.9 or 1.0% O_2_) to anaerobic (100% N_2_) cultures. When 20.9% O_2 _was introduced to an anaerobic culture, dissolved oxygen was present in the culture within less than 15 seconds and was >70% within less than 2 minutes. When 1.0% O_2 _was introduced to an anaerobic culture, dissolved oxygen was measurable in the culture within less than 2 minutes and for more than 2 h before returning to 0. When 20.9% O_2 _was replaced by N_2_, the dissolved oxygen decreased from ~80% to 0 within less than 2 minutes. No change in dissolved oxygen, which was 0, occurred when 1% O_2 _was replaced by N_2_.

The dilution rate and other conditions were maintained constant following a shift in conditions, but the differences in biomass yield in 0% (0.12 Cmol biomass [Cmol glucose]^-1^), 1.0% (0.36 Cmol biomass [Cmol glucose]^-1^) and 20.9% (0.60 Cmol biomass [Cmol glucose]^-1^) O_2 _[32] resulted in changes in specific growth rate for up to approximately 15 h following the shift. In cultures provided with either 20.9 or 1.0% O_2_, no increase in specific growth rate was observed during the first 2 h following the addition of O_2 _. Between 2 and approximately 10 h, cells in cultures receiving 20.9% O_2 _grew at 0.32 h^-1^, while those receiving 1.0% O_2 _grew at 0.21 h^-1^. After 10 h, growth continued at 0.10 h^-1^. In cultures which were made anaerobic, the specific growth rate decreased to 0.06 h^-1 ^almost immediately after O_2 _was replaced with N_2 _and returned to 0.10 h^-1 ^after approximately 15 h [32]. During these time intervals the cultures also experienced changes in extracellular metabolite concentrations, with ethanol and glycerol concentrations increasing in cultures which became anaerobic [32] and decreasing in cultures which became aerobic or hypoxic. Glycerol consumption did not occur, but ethanol consumption was observed in aerobic cultures. Ethanol production continued in the hypoxic cultures.

Oscillations occurred in cultures which were maintained in steady state anaerobic conditions and then provided with 20.9% O_2 _approximately 25 h after O_2 _was provided. Fresh, exponentially growing cells were not added to prevent oscillations since the transcript levels in the added cells may have affected the overall results disproportionately.

### Transcriptional analysis

Transcriptional analysis was performed with the TRanscript analysis with aid of Affinity Capture (TRAC) assay described by Rautio *et al*. [59]. Total mRNA was extracted from 10 mL cell culture samples (10 – 50 mg DW) which had been rapidly frozen in liquid N_2 _and stored at -80°C. GeneScan-120LIZ size standard (Applied Biosystems, USA) was added to each sample to calibrate the separation of the detection probes by size. In addition, *in vitro *synthesized mRNA (MEGAscript transcription kit; Ambion, USA) of the *Escherichia coli traT *gene was added to each sample (1.5 fmol [100 μl]^-1^) so that the results for each probe in any analysis could be correlated to this internal standard, eliminating experimental variation in different hybridizations and samples. Probes were divided into 2 probe pools with 8 and 9 probes per pool. The identity of the probes was determined by the migration behaviour and the quantity by the peak area. Total polyA RNA was quantified from the cell lysates, after eluting the polyA RNA in dimethyl pyrocarbonate (DMPC) treated H_2_O, using the RiboGreen RNA quantification kit (Molecular Probes, the Netherlands). Individual mRNA expression levels are given as the standardised (using *traT *internal standard) amount per total polyA RNA.

### Probes for TRAC analysis

The probes used in the TRAC analyses are listed in Table 1. They were designed using the mathematical algorithms presented in Kivoja *et al*. [60]. The probes of *HXT1-5, HXT8-10*, *GAL2, RGT2 *and *SNF3 *were designed to hybridise to the 3' end of the coding regions. The coding regions of *HXT13 *and *HXT17 *as well as *HXT9 *and *HXT11 *have sequence similarity of 97%. Only the 5' ends of the coding region of these genes differ enough to enable the design of specific probes. *HXT6 *and *HXT7 *are 99% identical within the coding region, but differences can be found in the 3' flank of the genes. The flanking region of *HXT7 *is AT rich which leads to a melting temperature of the probe (50°C) that is lower than that of the other probes (62–71°C). *HXT15 *and *HXT16 *are identical within their coding regions and within 1 kb in both directions from the coding region, except one nucleotide. Therefore only one probe was designed which detected both of these genes.

**Table 1:** Probes used in the TRAC analysis

**Gene**	**Probe sequence**	**Position**^a^
*HXT1*	ATGGTCAGGTGGGCATTTGTTAACTTTAGCTAA	826
*HXT2*	TAACTCACCCCAAGAAGCGTTACCG	891
*HXT3*	ATACCAATGGCACCATATAACAAACAGTTACGACGTC	1145
*HXT4*	CTGGATCGTCTGCGCTGACCTTATTTGAAAGAGCAATAG	842
*HXT5*	ATCATACCCATTAGTGTACGTCTGAAC	1026
*HXT6*	CATCTTGCCATACAATATAAATCGTAAGGGTTCAT	+78
*HXT7*	GTATATATTAAAAACGTATTTACTTTTCAAGATATCATTAAAA	+83
*HXT8*	AGAAGGGTTTCTCGTCATGCTGTAATTTTTCGT	1661
*HXT9*	ATGCTCAGTTTTTACAGATGGTGCATTTGCTACTGAG	60
*HXT10*	TGGCCAAAGACCTTCTAGCTTCTTCATACTTACCTTTTTCTAC	742
*HXT11*	GGCTCATTGGCATCTAGGTTAAGGGAATT	109
*HXT13*	ATCTCGAACATCTCCATCGCTATCAATAGAGGATT	14
*HXT14*	TATGGCCCTCGTTCTTAGAGGGAACAATTCG	1386
*HXT15/16*	GCCCATGTCGTTGCAAAGCAGAATATATAGAAGCATGTG	1284
*HXT17*	GACCATCCTGAATATCTCTATCACT	19
*SNF3*	TCTGTACTAGTAGGAATATCAACACGTTCTG	1892
*RGT2*	TTCACTTGTTTTGAAACAATCTAAAAGTGTTGACGGGCCGA	1007
*GAL2*	AGAGGCCAACGCCATACATTTCGACTTGA	1397

^a ^The start position of the probe inside the coding region. + indicates that the probe sequence is on the 3' side of the stop codon and the position is given relative to the stop codon.

### Sequencing of promoter regions

The following oligonucleotides were used in PCR of promoter fragments from the genomic DNA of CEN.PK113-1A: *HXT3 *promoter forward1 5'ACCGGTATATCAAATGGCGGTGTA 3', *HXT3 *promoter reverse 5'TCAGGCATGTTCATTACCTGAGAG 3', *HXT6 *promoter forward1 5'TGGCATCAAATTTGGGAA 3', *HXT6 *promoter reverse 5'GAGAGATGCTCCACAGGA 3', *HXT13 *promoter forward1 5'TGCTGCAATTTGCTATTT 3', *HXT13 *promoter reverse 5'CATCTCCATCGCTATCAA 3', *HXT15 *promoter forward1 5'CCATTTTTTCAGAATCCT 3', *HXT15 *promoter reverse 5'CTGTTTAGATTATCTGCA 3'. Three parallel PCR-reactions for each of the *HXT *promoters sequenced were carried out using Dynazyme Ext or Phusion high fidelity polymerases (Finnzymes, Finland), and purified PCR-fragments were sequenced using the same oligonucleotides that were used in the PCR-reactions. In addition, the following oligonucleotides were used in the sequencing: *HXT3 *promoter forward2 5'GGAACATTCTAGCTCGTT 3', *HXT6 *promoter forward2 5'TACTTGGAAATTAATGTA 3', *HXT13 *promoter forward2 5'ATCATTTGTCGTGTTCCT 3' and *HXT15 *promoter forward2 5'CCAAATATCTTATACGTT 3'.

### Data analysis and graphs

Statistical analysis of the data was carried out using SPSS software (version 14.0, SPSS Inc, USA,). Analysis of variance (ANOVA) and Dunnett's T3 multiple comparison test were used for the comparative analysis of the data. All graphs were prepared using the R environment [61,62].

## Authors' contributions

ER, LR and MP conceived the study. ER carried out the transcriptional, promoter and statistical analyses, participated in the fermentations and drafted the manuscript. AT and MGW carried out the fermentations and MGW revised the manuscript. LR supervised the work and revised the manuscript. All authors read and approved the final manuscript.
